# Supplementary material for: Water scarcity hotspots travel downstream due to human interventions in the 20th and 21st century
Source: Nat Commun. 2017 Jun 15;8:15697. doi: 10.1038/ncomms15697 (PMC5481728; doi:10.1038/ncomms15697)
Supplement: Supplementary Information — Supplementary Figures, Supplementary Table, Supplementary Methods and Supplementary References [file ncomms15697-s1.pdf]

Supplementary Methods

Defining water scarcity

Water scarcity refers to the imbalance between water availability and the needs for water over a specific time period and for a certain region[1-3]. In this study we focus on blue water scarcity only (hereafter: water scarcity) although we acknowledge that water scarcity related to the green (agricultural, soil moisture), and deep blue (fossil groundwater) water sources[4] form a significant part of the global water scarcity issue and must not, ideally, be assessed separately. Sophisticated modelling approaches to combine green, blue and deep blue water sources into one indicator for water scarcity are, however, currently lacking[5]. The monthly time-scale of this study limits us to the use of the water fluxes (streamflow, (sub-)surface runoff, and baseflow) while we assume the storage components of the blue water availability (lakes, aquifers, sub-surface reservoirs), for which the absolute values are unknown, to be in equilibrium. Consequently, we underestimate the actual blue water availability and overestimate the water scarcity conditions in those areas that heavily rely on these storage components. We do take into account the non-renewable groundwater resources, however, as well as the non-conventional water resources (desalination), although an indirect manner. As the use of these water resources lowers the needs for water we subtract the availability of these resources from our water withdrawal estimates, following the methodology of Wada et al.[6,7].

In this study we applied the water scarcity index (WSI)[3], an often used indicator that estimates the ratio between water withdrawals and water availability[8-19]. Following Mekonnen et al.[16] we explicitly incorporate minimum environmental flow requirements when estimating water scarcity conditions. If $WSI_{i,m}$ values are > 1, i.e. if more than 100% of the available fresh water resources is being allocated for environmental or anthropogenic needs[16], water scarcity is said to occur, see equation 1:

$$WSI_{i,m} = \frac{WW_{i,m}}{Q_{i,m} - EF_{i,m}}, \quad (1)$$

where $WSI_{i,m}$ is the water scarcity index for cell i and month m, $WW_{i,m}$ is the total water withdrawal in cell i and month m minus the water available in non-renewable groundwater resources and from desalination plants, $Q_{i,m}$ the total water availability in cell i and month m, and $EF_{i,m}$ the environmental flow requirement.

Describing and characterizing water scarcity

A number of variables were used in this study to describe and characterize water scarcity and water scarcity events. With exposure to water scarcity, we refer here to the share of population (using 2010 values)[20] and/or land area that experiences water scarcity in a certain month, time period, or place. For the purpose of comparison, exposure is often expressed as a relative term, i.e. exposed population as

percentage of the total population, globally, or per river basin. To distinguish between individual river basins we used the DDM30 river basin map[21]. The average duration is the average length of water scarcity events, calculated as:

$$Average\ Duration_i = \frac{NMscar_i}{NEscar_i}$$

where *Average Duration*$_i$ is the average duration of water scarcity events in cell *i*, $NMscar_i$ is the number of months with WSI > 1 in cell *i*, and $NEscar_i$ is the number of water scarcity events in cell *i*.

Modelling water resources availability

Water availability was estimated over the period 1971-2010 using monthly runoff and discharge (0.5° x 0.5°) from an ensemble of five impact models (**Supplementary Table 1**): H08[22,23], LPJmL[24,25], MATSIRO[26] , PCR-GLOBWB[7,27] , and WaterGAP[28]. These impact models were forced with daily inputs from three observations-based historical climate data-sets (PGFv2, GSWP3, WFD/WFDEI) creating an ensemble of 15 combinations. The Princeton Global Meteorological Forcing Dataset, version 2 (PGFv2) is an update of the forcing described by Sheffield et al.[29]. The GSWP3 data-set was developed as part of the third phase of the Global Soil Wetness Project (GSWP) and is a century long (1901– 2010), high resolution, global climate data-set (http://hydro.iis.u-tokyo.ac.jp/GSWP3/). The WFD/WFDEI dataset was created by applying the WFD methodology to the newer ECMWF ERA-Interim reanalysis data[30]. We refer to the individual model references and to Müller Schmied et al.[28] for a comprehensive overview of the different historical climate data-sets. The HYDE 3 – MIRCA dataset[20,31,32], assembled following Fader et al.[33], was used in this study as reference dataset for the historical irrigation and/or cropland patterns.

The estimated monthly water availability (Q_i), consists of a portion of locally generated runoff and a share of incoming discharge from upstream cells being deducted with the total upstream water consumption[6]:

$$Q_{i,m} = Q_{loc,i,m} + \sum_{j=i+1}^{n} \left(Q_{j,m} - WC_m\right), \quad (3)$$

where $Q_{i,m}$ is the total water availability in cell *i* (m^3 month^{-1}) and month *m*, $Q_{loc,i,m}$ the local runoff generated in cell *i* and month *m* (m^3 month^{-1}), $Q_{j,m}$ the discharge entering cell *i* from all upstream cells (*n = 7 or 8 in case of internal sink*) *j* in month *m* (m^3 month^{-1}), and WC_m the total upstream water consumption in month *m* (m^3 month^{-1}), encompassing the agricultural (irrigation and livestock), domestic, and industrial (energy and manufacturing) sectors. Under the NHI run WC_m is equal to zero. Each GHM uses a different water demand allocation method to estimate the $Q_{j,m} - WC_m$ under the HI run (**Supplementary Table 1**). Whereas water is supplied from surface water (first) in LPJmL,

H08, and MATSIRO to fulfil the consumptive water needs, WaterGAP always fulfils the water demand from groundwater and only from surface water in case of enough river flow. PCR-GLOBWB, finally, uses a ratio between the simulated daily baseflow and the long-term average river discharge as a proxy to infer the readily available amount of renewable groundwater reserves that can be used to accommodate consumptive water needs. As a result, the net discharge (accounting for the consumptive water needs) entering cell *i* from all upstream cells varies across the models, not only due to differences in generation of discharge or the height of the modelled water demands, but also due to the differences in fulfilment of these water demands by surface water and/or groundwater.

Modelling environmental flow requirements

Using the total monthly water availability per cell under the NHI, we estimated the minimum environmental flow requirements using the variable monthly flow (VMF) methodology[34]. Instream or environmental flow requirements[35-38] (EFR) are a relatively young topic in the discipline of global modelling of fresh water resources and water scarcity, with Smakthin et al.[37,38] being among the first to include them in global-scale analyses of fresh water resources and basin closure. Smakthin et al.[37] presented a first attempt to estimate the volume of water required for the maintenance of freshwater-dependent ecosystems at the global scale, using a "combination of ecologically relevant low-flow and high-flow components related to river flow variability and estimated by conceptual rules from discharge time series simulated by the global hydrology model". Building further on their experience and results, various global studies[16,39,40] have recently used either a predefined reservation of water (20-70%) or a low-flow indicator (Q90) to take into account a reservation of fresh water for environmental purposes in their fresh water resources and/or water scarcity assessments. Integrating the concept of EFR further in the global modelling of fresh water resources, Pastor et al.[34] compared and tested different calculation methods for the estimation of EFR in global-scale simulations. In this analysis, the authors compared for 11 case studies the locally defined EFR (with methods being used ranging from: hydrological methods, hydraulic methods, habitat simulation methods; or holistic methods – e.g. expert knowledge) with EFR estimates using a global methodological approach[35-37]. On top of the existing three global methods, two new methods were defined by Pastor et al.[34] that are combinations of the global methods mentioned before: a purely non-parametric method, using the flow quantiles Q90 and Q50 to estimate EFR, and a parametric method (the variable monthly flow - VMF), following the natural variability of river discharge, but increasing the protection of fresh water ecosystems during the low-flow season. According to their validation exercise, the VMF method is one of the best global approaches describing minimum environmental flow requirements at local and regional scales[34]. Given its performance, flexibility, and applicability, we therefore adopted this EFR method in our study. We are, nevertheless, aware of the fact that the environmental flow requirements included in our analysis are relatively simple "conceptual hydrology-based rules-of-thumb for the assessment of bulk environmental water requirements in world river basins"[37], and that more detailed

environmental flow requirements could be incorporated when looking at the scale of individual rivers or basins. Following the approach for estimation of the EFR via the VMF method[34], we determined first the low-flow, medium-flow, and high-flow months, and subsequently we assessed their associated environmental flow requirements as:

$$\begin{cases} EF_{i,m} = 0.6 * MMF_{i,m} \text{ given that } MMF_{i,m} \leq 0.4 * MAF_i \\ EF_{i,m} = 0.3 * MMF_{i,m} \text{ given that } MMF_{i,m} > 0.8 * MAF_i \\ EF_{i,m} = 0.45 * MMF_{i,m} \text{ given that } 0.4 * MAF_i < MMF_{i,m} \leq 0.8 * MAF_i \end{cases} \quad (4)$$

where $EF_{i,m}$ are the environmental flow requirements in cell i and month m, $MAF_{i,m}$ is the mean annual flow in cell i, and $MMF_{i,m}$ is the mean monthly flow in cell i and month m.

Modelling water demand

The consumptive water demands that are used in this study to adjust the river flow under the HI run (consumption) and to assess water scarcity under the HI and NHI run (withdrawal) encompass water demands of the agricultural sector (irrigation and livestock), the industry sector (energy and manufacturing), and water demands for domestic use.

For the calculation of all sectoral water demand a number of socio-economic parameters (GDP, population density, livestock density, land use and land cover) are used. To estimate irrigation, livestock, and industrial water demand, hydro-climatological parameters are used as well. Livestock water demands are estimated by multiplying the historical gridded livestock counts with their species-specific water daily demands[7,41,42]. Domestic water demands were derived using a time-series regression by individual countries and regions using the drivers population and GDP per capita[7,22,23,26,41-45]. PCR-GLOBWB additionally considers total electricity production, energy consumption, and temperature[7,41]. National numbers of domestic water use are distributed to a 0.5◦ by 0.5◦ grid according to the gridded total population numbers for all models and technological change rates were considered. Industrial water demands represent water being used for electricity production and/or manufacturing[7,22,23,26,41-45]. Whereas H08 and PCR-GLOBWB based their estimates of industrial water demands on historical country-scale aggregates (electricity production and manufacturing combined) from the WWDR-II[46-48] dataset and the FAO-AQUASTAT[49] database respectively[7,22,23,41,43], WaterGAP simulates global thermoelectric water use using gridded power plant data whilst manufacturing water demand was simulated for each country using the GVA per country and year, a technological change factor, and a manufacturing structural water use intensity[42]. Irrigation water use, finally, was estimated generally multiplying the area equipped for irrigation with the utilization intensity of irrigated land, the total crop water requirements per unit of irrigated area and the efficiency of irrigation that accounts for the losses during water transport and application of

the irrigation method[41]. Here, the specific crop water requirements are driven by the hydro-climatic conditions (temperature, precipitation, potential evapotranspiration, soil moisture, crop-growth curves, length and timing of the crop-growth season), whilst irrigation efficiency, the area equipped for irrigation and the utilization intensity are merely determined by economic, technological and political factors [7,22-26,28,31,44,45,50-53]. Each of these models have, moreover, performed an extensive validation study on the ability of their water demand model/ modelling frameworks to reflect historical developments in water demands, using historical observations or reported values as validation datasets[7,22,23,41,42,44]. For a systematic comparison and overview of the water demand calculation methods applied in each of the impact models we further refer to Wada et al.[41] and the individual model references. Four (H08, MATSIRO, PCR-GLOBWB, WaterGAP) of the five impact models considered modelled water withdrawals for the non-irrigation sectors. To cover non-irrigation water withdrawals in LPJmL, we used an ensemble-mean value that reflects the non-irrigation water withdrawal estimates from the other models. All impact models modelled irrigation water consumption and withdrawals endogenously. All, but WaterGAP, used the dynamic HYDE 3/MIRCA dataset as base-map for the extent of irrigated areas and cropland. Present-day (year 2000) rainfed and irrigated areas from MIRCA[31], cropland and pasture extents from Ramankutty et al.[32,], and trends of agricultural land from HYDE 3[20] were combined following Fader et al.[33] to create a time-varying dataset with information on croplands and the extent of irrigated areas. **Supplementary Figure 15** visualizes the expansion of irrigation areas and irrigation water demand over the period 1971-2010. Under transient conditions, all impact models diminished the river flow with endogenously (H08, MATSIRO, PCR-GLOBWB, WaterGAP) and/or partially endogenously (LPJmL) calculated water consumption. In this study, we diminished the river flow with the actual irrigation water consumption since this is the amount of water that is actually consumed given the restricted availability of fresh water. The WSI was calculated, however, using the potential irrigation water withdrawals since this is the volume of water that should ideally be available in order to accommodate all irrigation water needs.

Supplementary Figures

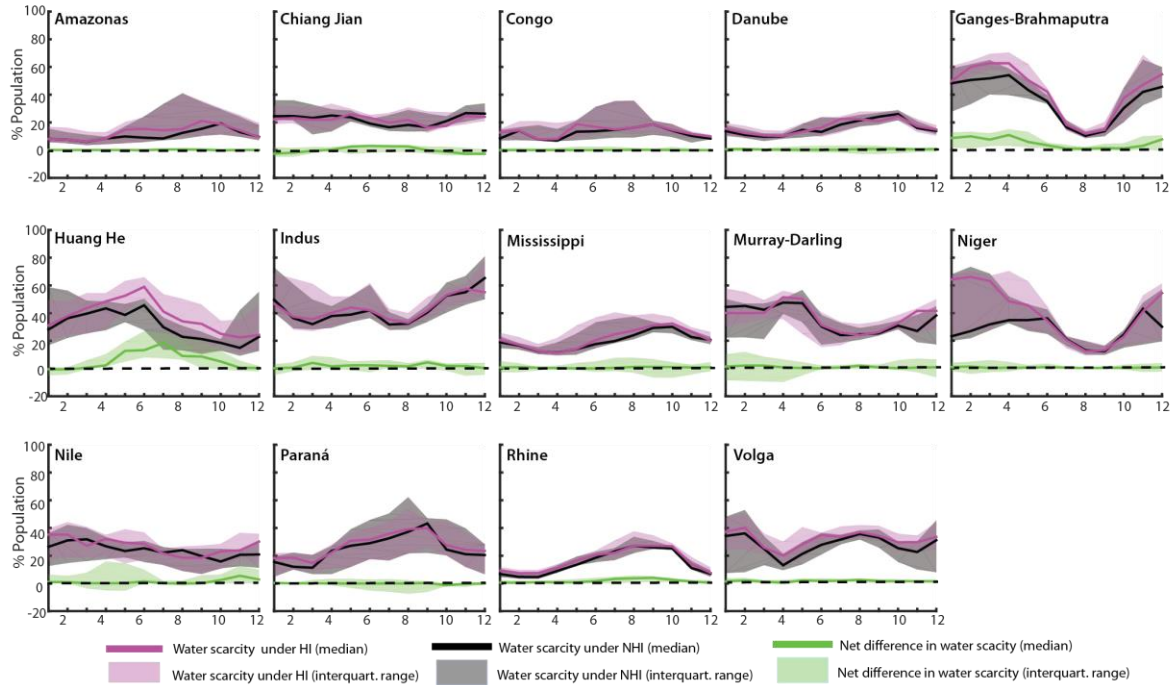

Supplementary Figure 1 - Seasonal exposure of population to water scarcity including and excluding human interventions. Supplementary Figure 1 shows for a selection of river basins the seasonal exposure to water scarcity, expressed as percentage of the population (2010). The magenta and black lines show the ensemble-median long-term mean exposure per month under the human interventions (HI) and no human interventions (NHI) run, respectively whilst the green line visualizes the long-term mean net monthly effect of HI on the exposure to water scarcity. The shaded areas show the interquartile range (q25-q75) in exposure to water scarcity and in the net impacts of HI on the exposure to water scarcity.

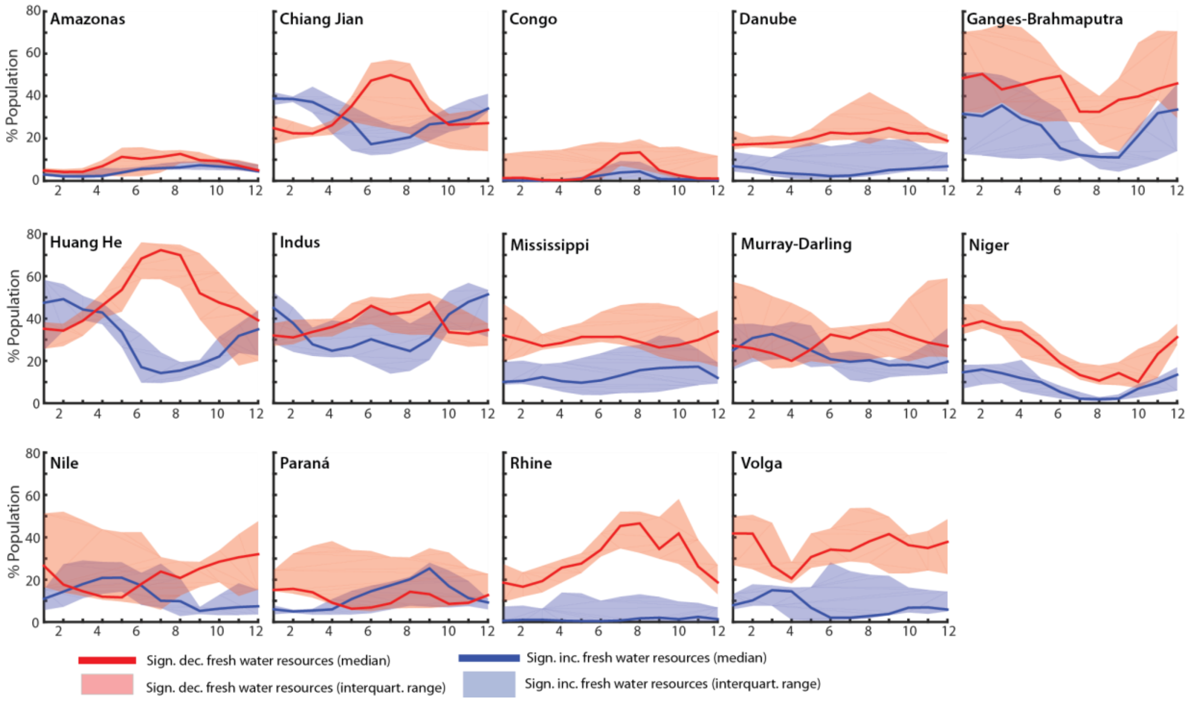

Supplementary Figure 2 –Seasonal exposure of population to significant changes in water availability caused by human interventions. Supplementary Figure 2 shows for a selection of river basins the seasonal exposure to significant changes in water availability due to human interventions (HI), expressed as percentage of the population (2010). Colored lines present the ensemble-median long-term mean exposure per month to any significant (>5%) decreases (red), and significant increases (blue) in water availability due to HI. The shaded areas visualize the interquartile ranges (q25-q75) around the ensemble-median results.

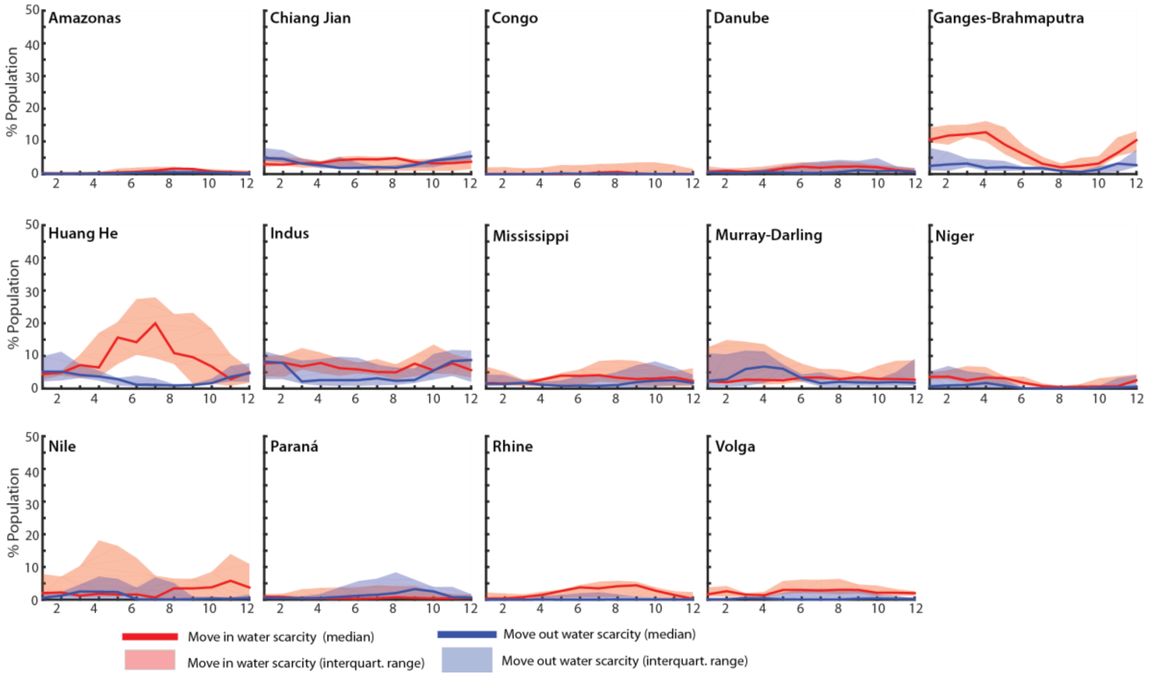

Supplementary Figure 3 – Seasonal share of population moving in/out of water scarcity due to human interventions. Supplementary Figure 3 shows for a selection of river basins per month the share of population that moves in/out of water scarcity due to human interventions (HI). Colored lines visualize the ensemble-median long-term mean population (%) that moves out (blue) or moves into (red) water scarcity, due to HI. The shaded areas visualize the interquartile ranges (q25-q75) around the ensemble-median results.

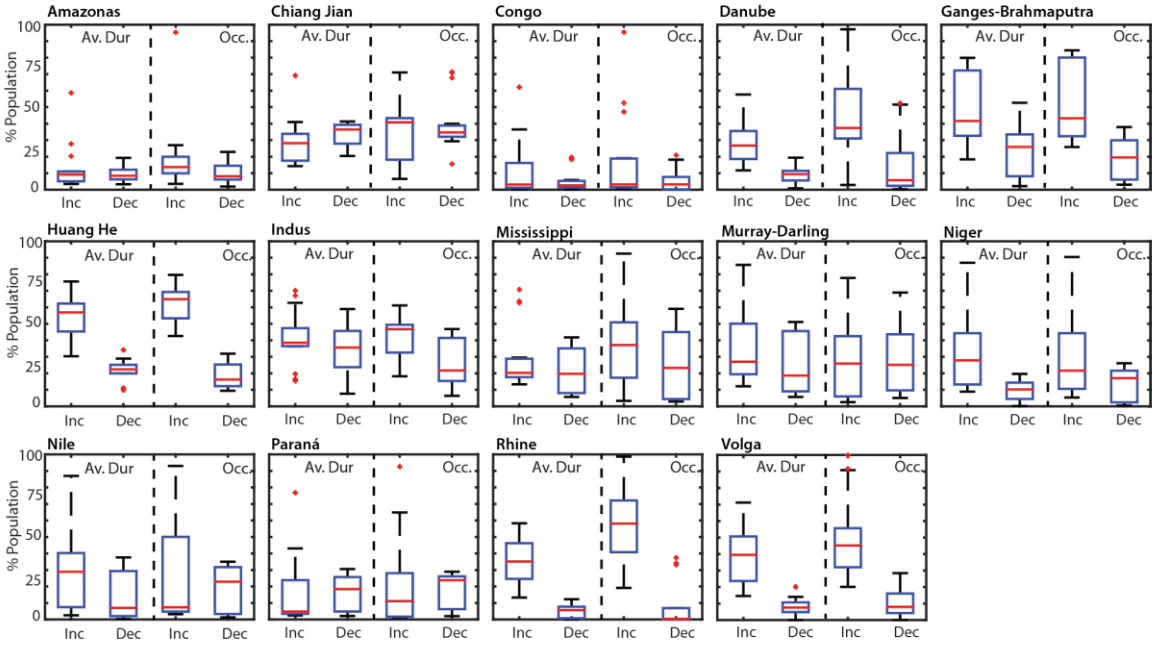

Supplementary Figure 4 – Share of population experiencing significant changes in average duration and occurrence of water scarcity events due to human interventions. Supplementary Figure 4 shows for a selection of river basins the share of population (2010 levels) experiencing significant increases or decreases in the average duration and occurrence of water scarcity events due to human interventions (HI). Boxplot values show the ensemble-median (red lines) as well as the interquartile ranges (blue boxes).

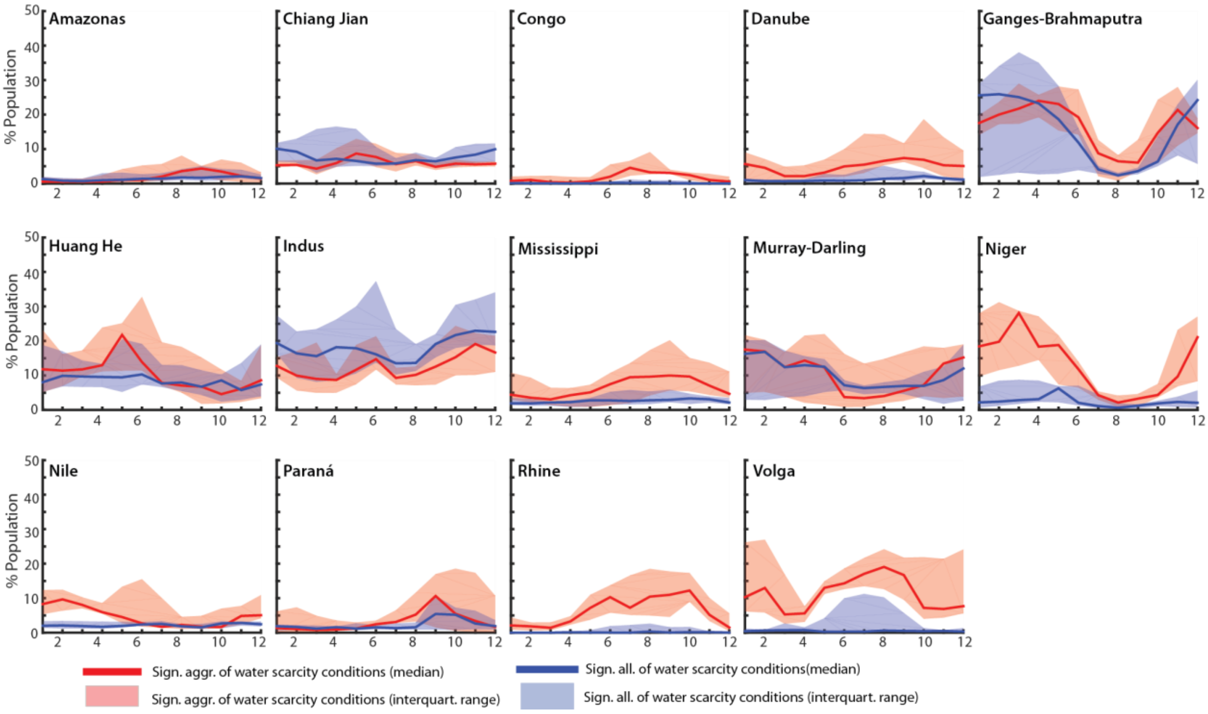

Supplementary Figure 5 – Share of population experiencing a significant alleviation/aggravation of water scarcity conditions due to human interventions. Supplementary Figure 5 shows for a selection of river basins the share of population experiencing a significant aggravation or alleviation in water scarcity conditions due to human interventions (HI). The colored lines visualize the ensemble-median long-term mean population (%) that experiences a significant (>5%) alleviation (blue) or aggravation (red) of water scarcity conditions, whilst already being in water scarce conditions, due to HI. The shaded areas visualize the interquartile ranges (q25-q75) around the ensemble-median results.

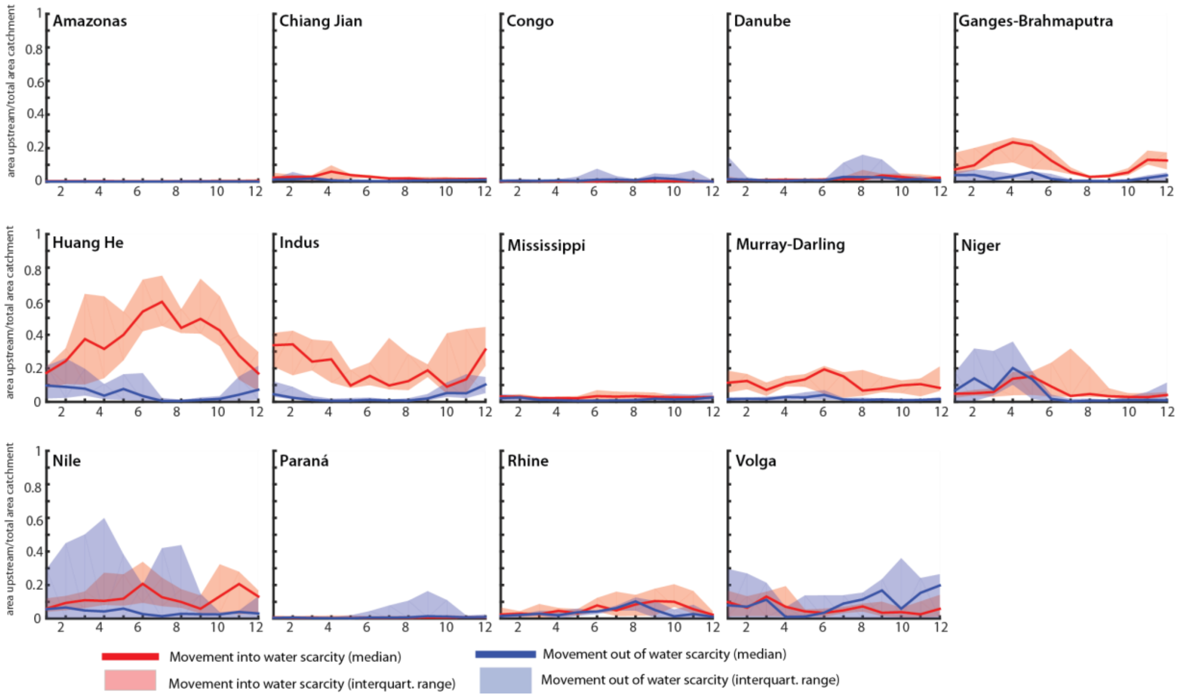

Supplementary Figure 6 - Population-weighted ratio between upstream area/total catchment area when looking at the movement into or out of water scarcity due to human interventions. Supplementary Figure 6 shows for a selection of basins the ratio between the upstream area and the total catchment area for areas, when selecting for those moving out of water scarcity due to human interventions (HI) (blue); and those moving into water scarcity due to HI (red). The shaded areas visualize the interquartile ranges (q25-q75) around the ensemble-median results.

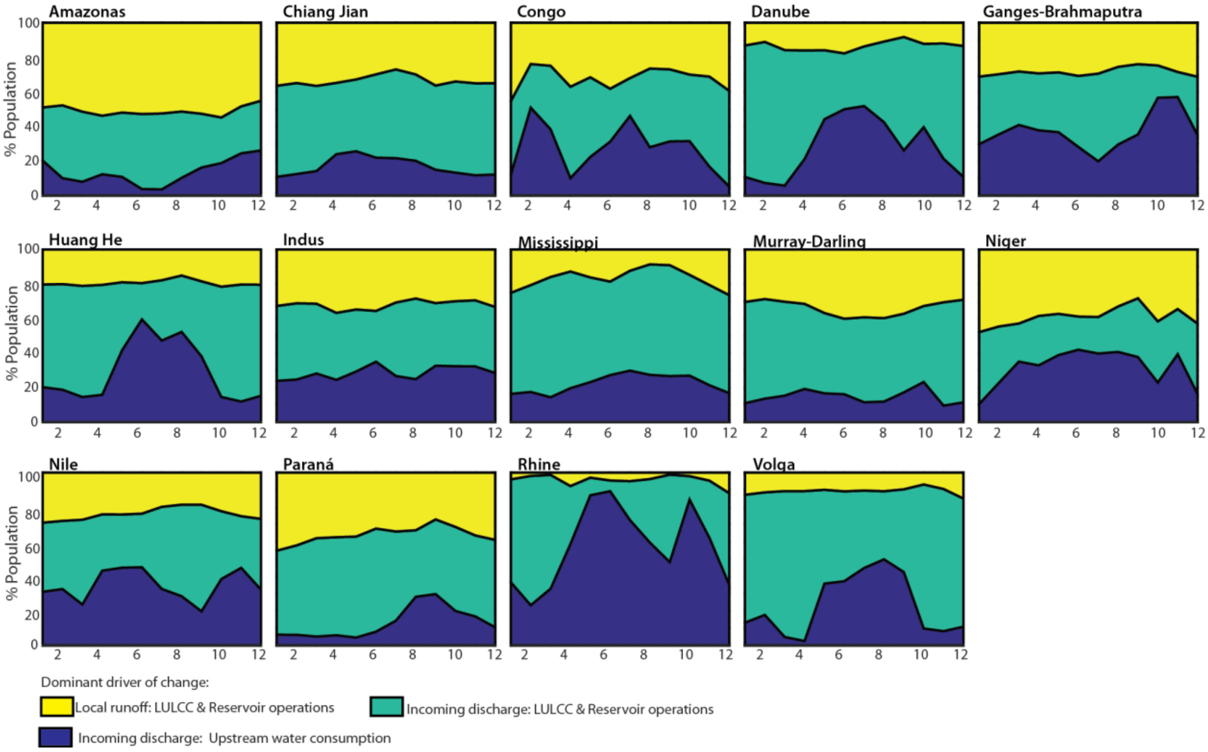

Supplementary Figure 7 - Dominant driver of changes in water availability due to human interventions. Supplementary Figure 7 shows the share of the global population (2010) having as dominant driver and origin of change: Local runoff: Land Use and Land Cover Change (LULCC) & Reservoirs; Incoming discharge: LULCC & Reservoirs; or Incoming discharge: Upstream water consumption, when taking into account all significant changes in water availability due to human interventions (HI).

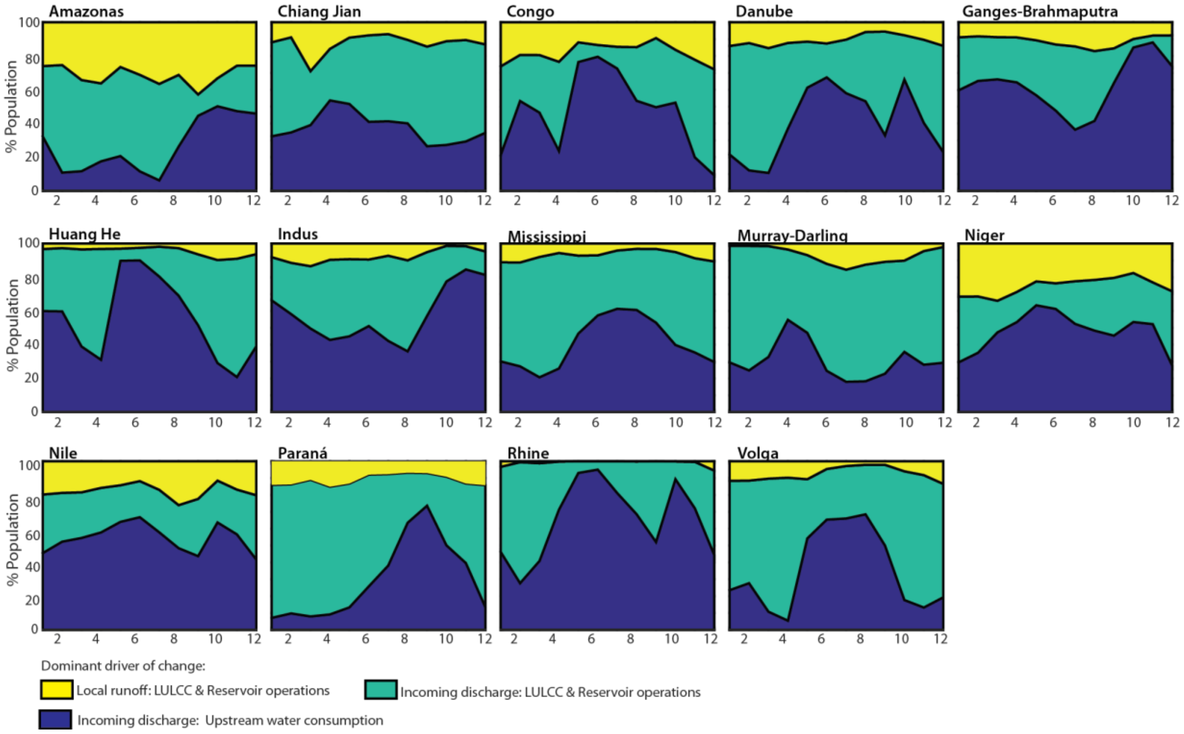

Supplementary Figure 8 - Dominant driver of decreases in water availability due to human interventions. Supplementary Figure 8 shows the share of the global population (2010) having as dominant driver and origin of change: Local runoff: Land Use and Land Cover Change (LULCC) & Reservoirs; Incoming discharge: LULCC & Reservoirs; or Incoming discharge: Upstream water consumption, when taking into account only those areas with significant decreases in water availability due to human interventions (HI).

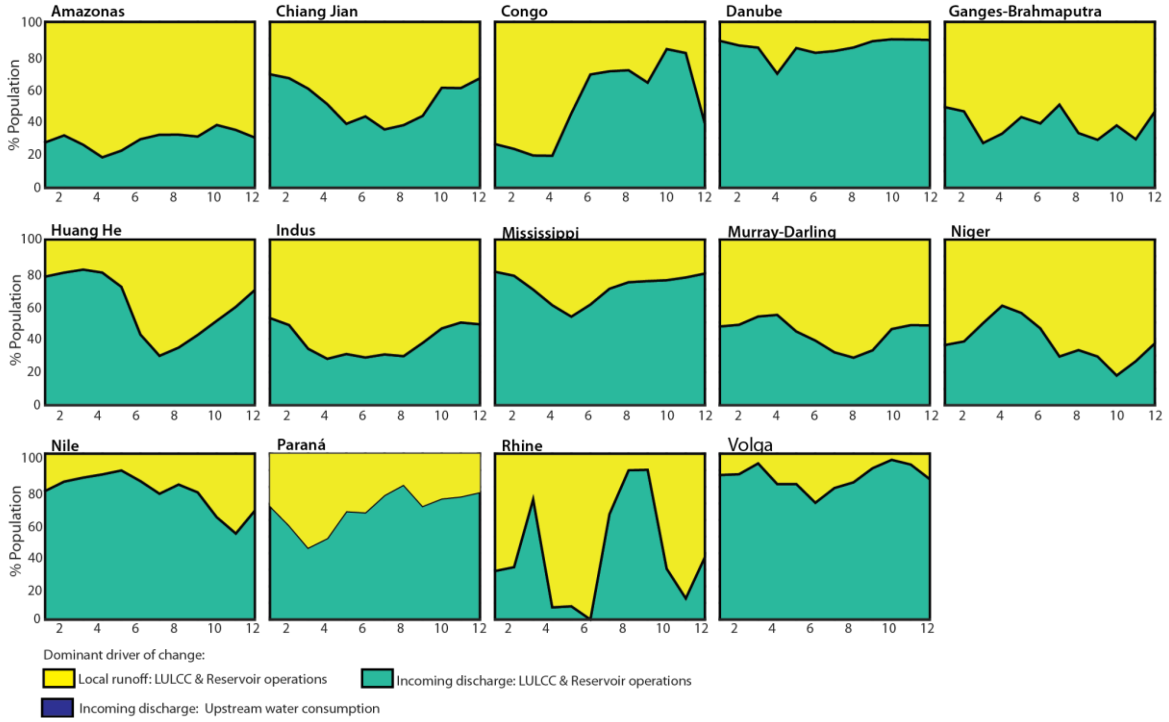

Supplementary Figure 9 - Dominant driver of increases in water availability due to human interventions. Supplementary Figure 9 shows the share of the global population (2010) having as dominant driver and origin of change: Local runoff: Land Use and Land Cover Changes (LULCC) & Reservoirs; Incoming discharge: LULCC & Reservoirs; Incoming discharge: Upstream water consumption, when taking into account only those areas with significant increases in water availability due to human interventions (HI).

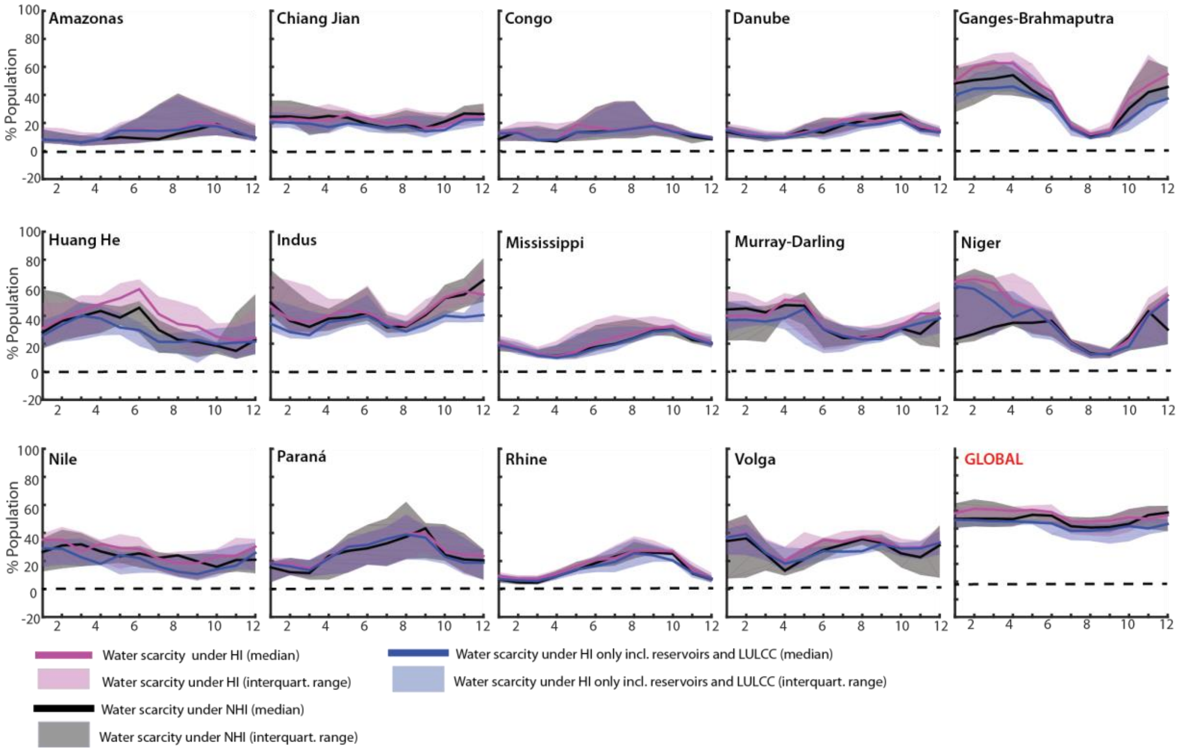

Supplementary Figure 10 - Seasonal exposure of population to water scarcity with and without reservoir operations and land use and land cover change as only human interventions. Supplementary Figure 10 shows for a selection of river basins and globally the seasonal exposure to water scarcity, expressed as percentage of the population (2010). The magenta and black lines show the ensemble-median long-term mean exposure per month under the human interventions (HI) and no human interventions (NHI) run, respectively. The blue line visualizes the ensemble-median long-term mean exposure per month when taking into account only reservoir operations and Land Use and Land Cover Change (LULCC) as HI. The shaded areas show the interquartile range (q25-q75) in exposure to water scarcity.

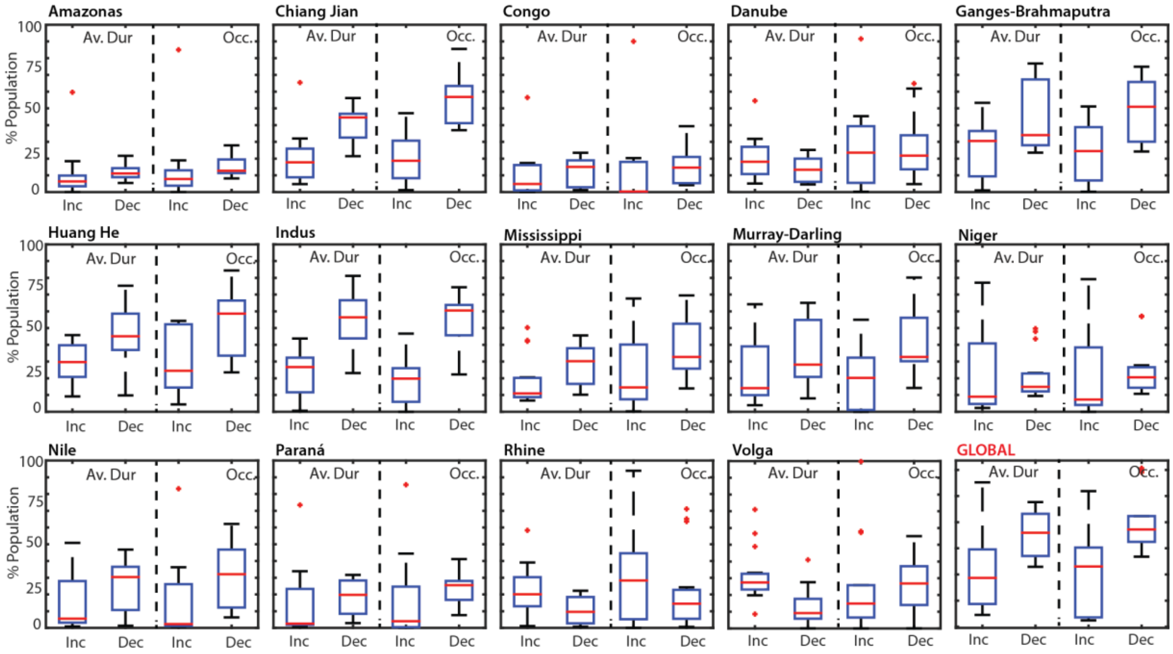

Supplementary Figure 11 – Share of population experiencing significant changes in average duration and occurrence of water scarcity events due to implementation of reservoir operations and land use and land cover change as only human intervention. Supplementary Figure 11 shows for a selection of river basins and globally the share of population (2010 levels) exposed to significant increases or decreases in the average duration of water scarcity events and occurrence of water scarcity due to the implementation of reservoir operations and Land Use and Land Cover Change (LULCC). Boxplot values show the ensemble-median (red lines) as well as the interquartile ranges (blue boxes).

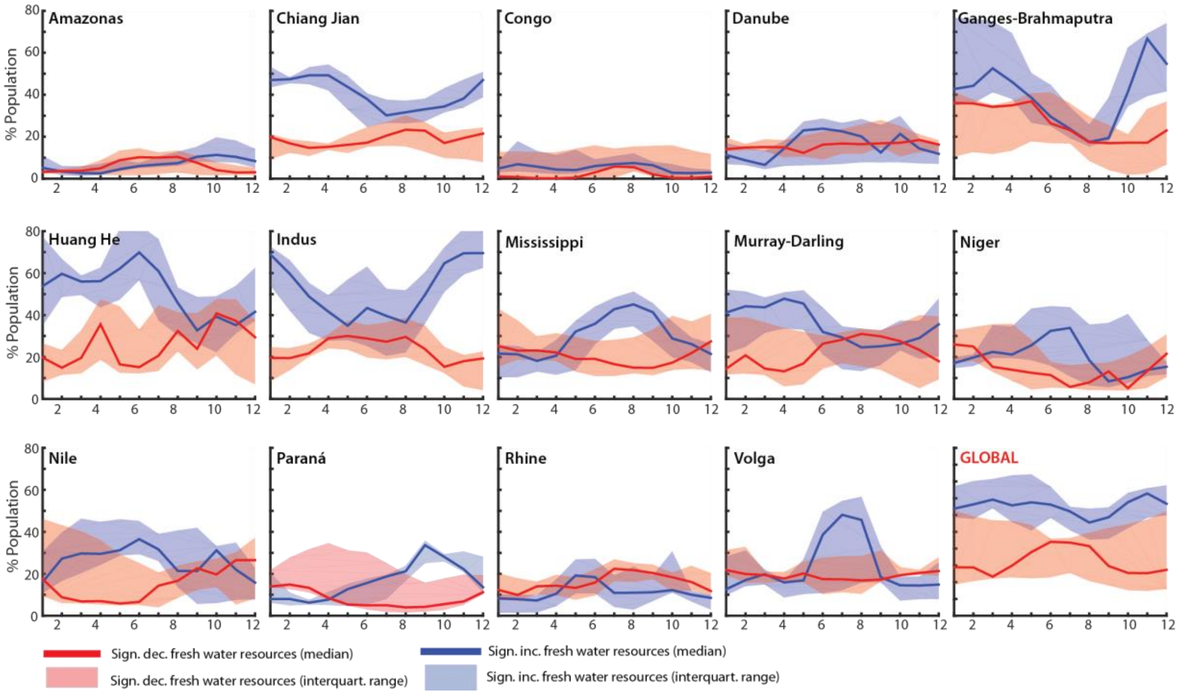

Supplementary Figure 12 – Exposure of population to significant changes in water availability due to implementation of reservoir operations and land use and land cover change as only human intervention. Supplementary Figure 12 shows for a selection of river basins and at the global scale the seasonal exposure to significant changes in water availability due to implementation of reservoir operation and Land Use and Land Cover Change (LULCC), expressed as percentage of the population (2010). Colored lines present the ensemble-median long-term mean exposure per month to any significant (>5%) decreases (red), and significant increases (blue) in water availability. The shaded areas visualize the interquartile ranges (q25-q75) around the ensemble-median results.

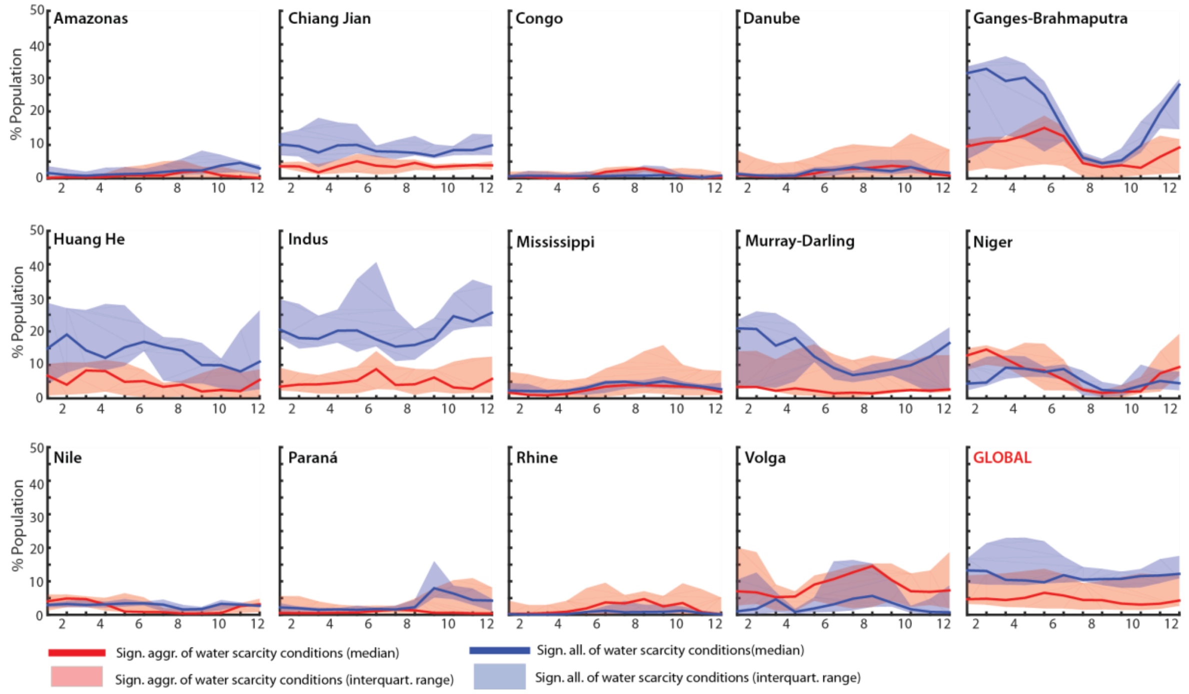

Supplementary Figure 13 – Share of population being exposed to significant aggravation or alleviation of water scarcity conditions due to implementation of reservoir operations and land use and land cover change as only human intervention. Supplementary Figure 13 shows for the global scale and a selection of river basins the seasonal exposure to significant changes in water scarcity conditions due to implementation of reservoir operation and Land Use and Land Cover Change (LULCC), expressed as percentage of the population. Colored lines visualize the ensemble-median long-term mean population (%) that experiences a significant (>5%) alleviation (blue) or aggravation (red) of water scarcity conditions, whilst already being in water scarce conditions. The shaded areas visualize the interquartile ranges (q25-q75) around the ensemble-median results.

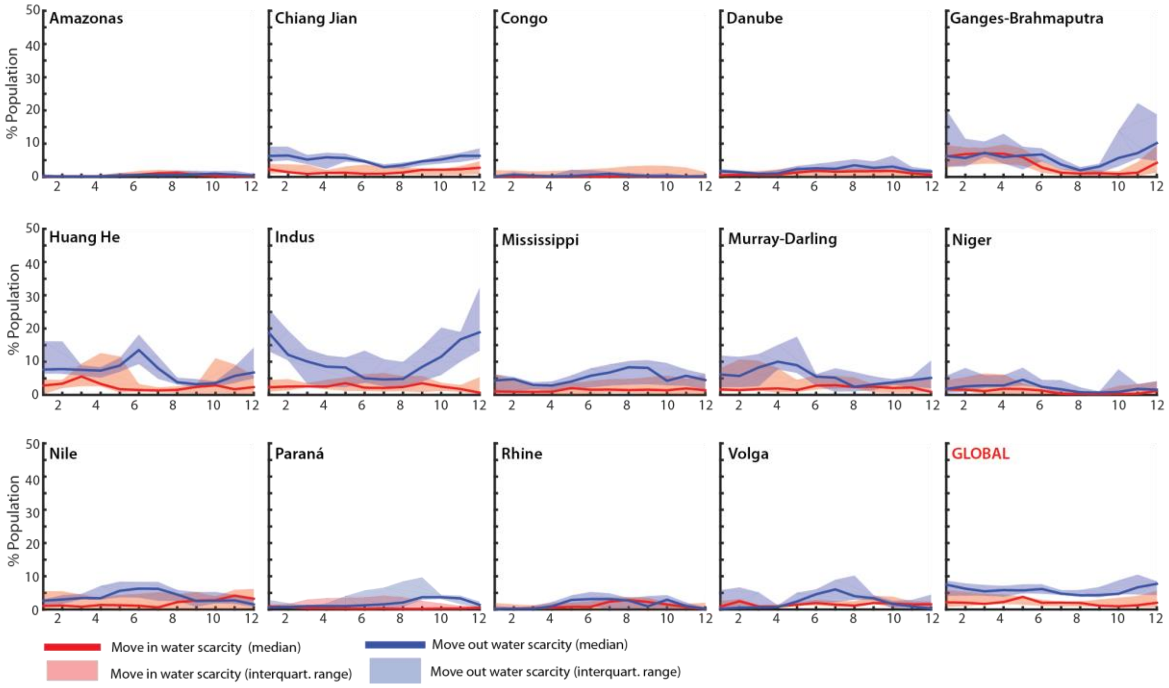

Supplementary Figure 14 – Exposure of population to movement in or out of water scarcity due to implementation of reservoir operation and land use and land cover change as only human intervention. Supplementary Figure 14 shows for a selection of river basins and globally per month the share op population that moves in/out of water scarcity due to implementation of reservoir operations and Land Use and Land Cover Change (LULCC). Colored lines visualize the ensemble-median long-term mean population (%) that moves out (blue) or moves into (red) water scarcity. The shaded areas visualize the interquartile ranges (q25-q75) around the ensemble-median results.

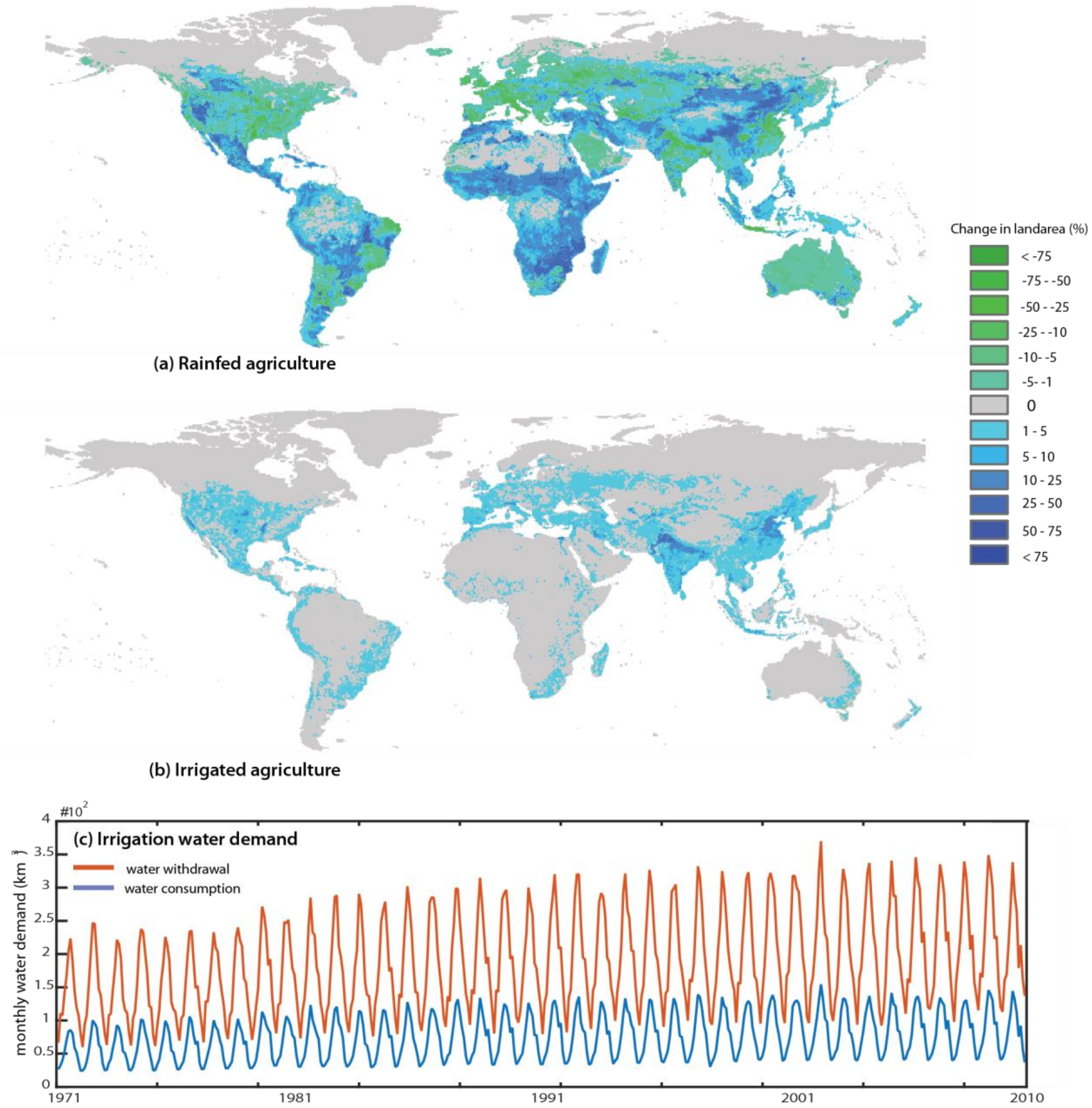

Supplementary Figure 15 – Expansion of agricultural land area and water demands over the period 1971-2010. Figure 15a shows the change in rainfed agricultural land area over the period 1971-2010; Figure 15b shows the expansion of irrigated agriculture over the period 1971-2010; Figure 15c shows the expansion and seasonality in irrigation water demand (withdrawals and consumption) over the period 1971-2010.

Supplementary Tables

Supplementary Table 1 –Model characteristics

Model name	Time step	Spatial resolu-tion	Meteorol ogical forcing	Forcing datasets	Evapora-tion cheme	Runoff scheme	Snow scheme	Irrigated area and/or Crop area	Irrigati on water demand	Non-Irrigation water demand	Water demand allocation	References
H08	Daily	0.5º	R, S, T, W, Q, LW, SW, SP	PGMFD v.2, GSWP3, WFD/WFDEI	Bulk formula	Saturation excess, non-linear	Energy balance	Time-varying HYDE 3/ MIRCA	Yes	Domestic, Manufacturing	For ISIMIP2a simulations, water is supplied from rivers	Hanasaki et al.[22,23]
LPJmL	Daily	0.5º	P, T, LWn, SW	PGMFD v.2, GSWP3, WFD/WFDEI	Priestley-Taylor	Saturation excess	Degree-day	Time-varying HYDE 3/ MIRCA	Yes	None*	LPJmL fulfils all water demand from surface water (incl. reservoirs), first from within the cell, then from neighbouring cells. In case of actual water demands, withdrawals are limited to surface water availability.	Bondeau et al.[24] Schaphoff et al.[25]
MATSIRO	1 hr	0.5º	R, S, T, W, Q, LW, SW, SP	PGMFD v.2, GSWP3, WFD/WFDEI	Bulk formula	Infiltration excess, saturation excess, groundwater	Energy balance	Time-varying HYDE 3/ MIRCA	Yes	Domestic, Industry, Livestock	Surface water is withdrawn first (until rivers are depleted to the threshold set by environmental flow requirement), and the unfulfilled demand is then taken from groundwater.	Pokhrel et al. [26]
PCR-GLOBWB	Daily	0.5º	P,T	PGMFD v.2, GSWP3, WFD/WFDEI	Hamon	Saturation excess beta function	Degree-day	Time-varying HYDE 3/ MIRCA	Yes	Domestic, Industry, Livestock	Since the absolute amount of available groundwater resources is not known at the global scale,PCR-GLOBWB uses the ratio between the simulated daily (accumulated) baseflow against the long-term average river discharge as a proxy to infer the readily available amount of renewable groundwater reserves	Van Beek et al.[27] Wada et al.[7]
WaterGAP	Daily	0.5º	P, T, LW, SW	PGMFD v.2, GSWP3, WFD/WFDEI	Priestley-Taylor	Beta function	Degree-day	Time-varying HYDE 3/ MIRCA	Yes	Domestic, Manufacturing, Electricity production, Livestock	Always fulfil water demand from groundwater but from surface water only in case of enough river water	Müller Schmied et al.[28]

*LPJmL does not estimate non-irrigation water demands itself. In this study we used an ensemble-mean value that reflects the non-irrigation water demands from the other models applied.

Supplementary References

1. Falkenmark, M., Lundqvist, J. & Widstrand, C. Macro-scale water scarcity requires micro-scale approaches: Aspects of vulnerability in semi-arid development. *National Resources Forum* **13**, 258–267 (1989).

2. Falkenmark, M. Growing water scarcity in agriculture: future challenge to global water security. *Philosophical Transactions of the Royal Scoiety A: Mathematical, Physical and Engineering Sciences* **371**, 20120410 (2013).

3. Raskin, P., Gleick, P., Kirshen, P., Pontius, G. & Strzepek, K. Comprehensive assessment of the freshwater resources of the world. Stockholm Environment Institute, Stockholm, Sweden (1997).

4. Savenije, H.H.G. Water scarcity indicators; the deception of the numbers. *Phys. Chem. Earth (B)* **25**, 199-204 (2000).

5. Schyns, J. F., Hoekstra, A. Y. & Booij, M. J. Review and classification of indicators of green water availability and scarcity. *Hydrol. Earth. Syst. Sci.* **19**, 4581-4608 (2015).

6. Wada, Y., Van Beek, L.P.H. & Bierkens, M.F.P. Modelling global water stress of the recent past: on the relative importance of trends in water demand and climate variability. *Hydrol. and Earth Syst. Sci.* **15**, 3785–3808 (2011).

7. Wada, Y., Wisser, D. & Bierkens, M.F.P. Global modelling of withdrawal, allocation and consumptive use of surface water and groundwater resources. *Earth Syst. Dyn.* **5** 15–40 (2014).

8. Alcamo, J. *et al*. Global estimates of water withdrawals and availability under current and future 'business-as-usual' conditions. *Hydrol. Sci.—J.* **48**, 339–348 (2003).

9. Alcamo, J., Flörke, M., & Märker, M. Future long-term changes in global water resources driven by socio-economic and climatic changes. *Hydrol. Sci.—J.* **52**, 247–275 (2007).

10. Arnell, N. W. Climate change and global water resources. *Env.Change* **9**, S31–S49 (1999).

11. Gosling, S.N. & Arnell N.W. A global assessment of the impact of climate change on water scarcity. *Clim. Change* **134**, 371-385 (2016).

12. Hanasaki, N. *et al*. A global water scarcity assessment under Shared Socio-economic Pathways – Part 2: Water availability and scarcity. *Hydrol. Earth Syst. Sci.* **17**, 2393-2413 (2013).

13. Hoekstra, A.Y., Mekonnen, M.M., Chapagain, A.K., Mathews, R.E., & Richter, B.D. Global monthly water scarcity: blue water footprints versus blue water availability. *PloS One* **7**, e32688 (2012).

14. Kiguchi, M., Shen, Y., Kanae, S. & Oki, T. Reevaluation of future water stress due to socio-economic and climate factors under a warming climate. *Hydrol. Sci.—J.* **60**, 14-29 (2015).

15. Kundzewicz, Z.W. *et al*. The implications of projected climate change for freshwater resources and their management. *Hydrol. Sci.—J.* **53**, 3–10 (2008).

16. Mekonnen, M.M., & Hoekstra, A.Y. Four billion people facing severe water scarcity. *Sci. Adv.* **2**, e1500323 (2016).

17. Veldkamp, T.I.E., Eisner, S., Wada, Y., Aerts, J.C.J.H. & Ward, P.J. Sensitivity of water scarcity events to ENSO driven climate variability at the global scale. *Hydrol. and Earth Syst. Sci*. **19**, 4081-4098 (2015a)

18. Veldkamp, T.I.E., *et al.* Changing mechanism of global water scarcity events: Impacts of socioeconomic changes and inter-annual hydro-climatic variability. *Glob. Env. Change* **32,** 18–29 (2015b).

19. Vörösmarty, C.J., Green, P., Salisbury, J. & Lammers, R.B. Global Water Resources: Vulnerability from Climate Change and Population Growth. *Science* **289**, 284–288 (2000).

20. Klein Goldewijk, K. & Van Drecht, G. HYDE 3: Current and historical population and land cover. Integrated modelling of global environmental change. An overview of IMAGE 2.4. (Eds: A.F. Bouwman, T. Kram and K. Klein Goldewijk)Netherlands Environmental Assessment Agency (MNP), Bilthoven, The Netherlands (2006).

21. Döll, P. & Lehner, B. Validation of a new global 30-min drainage direction map. *Journal of Hydrology* **258**, 214-231 (2002).

22. Hanasaki, N. *et al*. An integrated model for the assessment of global water resources – Part 1: Model description and input meteorological forcing. *Hydrol. Earth Syst. Sci.***12**, 1007–1025 (2008a).

23. Hanasaki, N. *et al*. An integrated model for the assessment of global water resources – Part 2: Applications and assessments. *Hydrol. Earth Syst. Sci.* **12**, 1027–1037 (2008b).

24. Bondeau, A. *et al.* Modelling the role of agriculture for the 20th century global terrestrial carbon balance. *Global Change Biology* **13**, 679–706 (2007).

25. Schaphoff, S. *et al.* Contribution of permafrost soils to the global carbon budget. *Env. Res. Lett*. **8**, 014026 (2013).

26. Pokhrel, Y.N. *et al*. Incorporation of groundwater pumping in a global land surface model with the representation of human impacts, *Water Resour. Res*. **51**, 78–96 (2015).

27. Van Beek, L.P.H., Wada, Y. and Bierkens, M.F.P. Global monthly water stress: I. Water balance and water availability. *Water Resour. Res.* **47**, W07517 (2011).

28. Müller Schmied, H. *et al.* Variations of global and continental water balance components as impacted by climate forcing uncertainty and human water use. *Hydrol. Earth Syst. Sci*. **20**, 2877-2898 (2016).

29. Sheffield, J., Goteti, G. & Wood, E.F. Development of a 50-yr high-resolution global dataset of meteorological forcings for land surface modelling. *J. Climate* **19**, 3088-3111 (2006).

30. Weedon, G.P. *et al*. The WFDEI meteorological forcing data set: WATCH Forcing Data methodology applied to ERA-Interim reanalysis data. *Water Resour. Res*. **50**, 7505-7514 (2014).

31. Portmann, F.T., Siebert, S. & Döll., P. MIRCA2000 – Global monthly irrigated and rainfed crop areas around the year 2000: A new high-resolution data set for agricultural and hydrological modelling. *Global Biogeochem. Cycles* **24**, GB1011 (2010).

32. Ramankutty, N., Evan, A.T., Monfreda, C. & Foley, J.A. Farming the planet: 1. Geographic distribution of global agricultural lands in the year 2000. *Global Biogeochem. Cycles* **22**, GB1003 (2008).

33. Fader, M., Rost, S., Müller, C., Bondeau, A. & Gerten, D. Virtual water content of temperate cereals and maize: Present and potential future patterns. *Journal of Hydrology* **384**, 218-231 (2010).

34. Pastor, A.V., Ludwig, F., Biemans, H., Hoff, H. & Kabat, P. Accounting for environmental flow requirements in global water assessments. *Hydrol. Earth. Syst. Sci.* **18**, 5041-5059 (2014).

35. Tessmann, S. Environmental assessment, technical appendix e in environmental use sector reconnaissance elements of the western dakotas region of south dakota study. South dakota state university, Water Resources Institute, South Dakota State University, Brookings, South Dakota (1980).

36. Tennant, D.L. Instream flow regimens for fish, wildlife, recreation and related environmental resources. *Fisheries* **1**, 6–10 (1976).

37. Smakhtin, V., Revenga, C., and Döll, P. A pilot global assessment of environmental water requirements and scarcity. *Water Int.* **29**, 307–317 (2004).

38. Smakhin, V. Basin closure and environmental flow requirements. *Internat. J. of Wat. Res. Dev.* **24**, 227-233 (2008).

39. Wada, Y., Van Beek, L.P.H., Wanders, N. & Bierkens, M.F.P. Human water consumption intensifies hydrological drought worldwide. *Env. Res. Lett.* **8**, 034036 (2013).

40. Bonsch, M., et al. Environmental flow provision: Implications for agricultural water and land-use at the global scale. Glob. Env. Change, 30, 113-132 (2015).

41. Wada, Y. *et al.* Modeling global water use for the 21st century: Water Futures and Solutions (WFaS) initiative and its approaches. *Geosci. Model Dev.* **9**, 175-222 (2016).

42. Flörke, M. *et al.* Domestic and industrial water uses of the past 60 years as a mirror of socio-economic development: A global simulation study. *Global Environ. Change* **23**, 144–156 (2013).

43. Yoshikawa, S., Cho, J., Yamada, H.G., Hansaki, N. & Kanae, S. An assessment of global net irrigation water requirements from various water supply sources to sustain irrigation: rivers and reservoirs (1960-2050). *Hydrol. Earth Syst. Sci*. **18**, 4289-4310 (2014).

44. Pokhrel, Y.N. *et al.* Incorporating anthropogenic water regulation modules into a land surface model. *J Hydrometeor.* **13**, 255–269 (2012).

45. Takata, K., Emori, S. & Watanabe, T. Development of minimal advanced treatments of surface interaction and runoff. *Global Planet. Change* **38**, 209–222 (2003).

46. Shiklomanov, I. A. Assessment of water resources and water availability in the world, Comprehensive assessment of the freshwater resources of the world, World Meteorological Organization and the Stockholm Environment Institute (1997).

47. World Resources Institute. World Resources: A Guide to the Global Environment 1998–99, World Resources Institute, Washington DC, USA (1998).

48. Vörösmarty, C.J., Leveque, C. & Revenga, C. Millennium Ecosystem Assessment Volume 1: Conditions and Trends, chap. 7: Freshwater ecosystems. Island Press, Washington DC, USA, 165–207 (2005).

49. FAO – Food and Agriculture Organization of the United Nations. AQUASTAT online database, available at: http://www.fao.org/nr/water/aquastat/dbase/index.stm, last access: 16th January 2017.

50. Döll, P. & Siebert, S. Global modelling of irrigation water requirements. *Water Resour. Res* **38**, W1037 (2002).

51. Döll, P. & Müller Schmied, H. How is the impact of climate change on river flow regimes related to the impact on mean annual runoff? A global-scale analysis. *Environ. Res. Lett.* **7**, 014037 (2012).

52. Döll, P., Fritsche, M., Eicker, A. & Müller Schmied, H. Seasonal Water Storage Variations as Impacted by Water Abstractions: Comparing the Output of a Global Hydrological Model with GRACE and GPS Observations. *Surv. Geophys*. **35**, 1311-1331 (2014).

53. Müller Schmied, H. *et al.* Sensitivity of simulated global-scale freshwater fluxes and storages to input data, hydrological model structure, human water use and calibration. *Hydrol. Earth. Syst. Sci*. **18**, 3511-3538 (2014).
